# Prevalence of physical activity counseling in primary care: A systematic review and meta-analysis

**DOI:** 10.34172/hpp.2023.31

**Published:** 2023-12-16

**Authors:** Apichai Wattanapisit, Sarawut Lapmanee, Sirawee Chaovalit, Charupa Lektip, Palang Chotsiri

**Affiliations:** ^1^Department of Clinical Medicine, School of Medicine, Walailak University, Nakhon Si Thammarat, Thailand; ^2^Family Medicine Clinic, Walailak University Hospital, Nakhon Si Thammarat, Thailand; ^3^Department of Basic Medical Sciences, Faculty of Medicine, Siam University, Bangkok, Thailand; ^4^Department of Physical Therapy, Faculty of Medicine, Prince of Songkla University, Songkhla, Thailand; ^5^Department of Physical Therapy, School of Allied Health Sciences, Walailak University, Nakhon Si Thammarat, Thailand; ^6^Department of Clinical Pharmacology, Mahidol-Oxford Tropical Medicine Research Unit, Faculty of Tropical Medicine, Mahidol University, Bangkok, Thailand

**Keywords:** Counseling, Exercise, Meta-analysis, Prevalence, Primary health care

## Abstract

**Background:**

This systematic review aimed to summarize and evaluate the prevalence of physical activity (PA) counseling in primary care.

**Methods:**

Five databases (CINAHL Complete, Embase, Medline, PsycInfo, and Web of Science) were searched. Primary epidemiological studies on PA counseling in primary care were included. The Joanna Briggs Institute critical appraisal checklist for studies reporting prevalence data was used to assess the quality of studies. The review protocol was registered with PROSPERO (CRD42021284570).

**Results:**

After duplicate removal, 4990 articles were screened, and 120 full-text articles were then assessed. Forty studies were included, with quality assessment scores ranging from 5/9 to 9/9. The pooled prevalence of PA counseling based on 35 studies (199830 participants) was 37.9% (95% CI 31.2 to 44.6). The subgroup analyses showed that the prevalence of PA counseling was 33.1% (95% CI: 22.6 to 43.7) in females (10 studies), 32.1% (95% CI: 22.6 to 41.7) in males (10 studies), 65.5% (95% CI: 5.70 to 74.1) in people with diabetes mellitus (6 studies), 41.6% (95% CI: 34.9 to 48.3) in people with hypertension (5 studies), and 56.8% (95% CI: 31.7 to 82.0) in people with overweight or obesity (5 studies). All meta-analyses showed high levels of heterogeneity (I^2^=93% to 100%).

**Conclusion:**

The overall prevalence of PA counseling in primary care was low. The high levels of heterogeneity suggest variability in the perspectives and practices of PA counseling in primary care. PA counseling should be standardized to ensure its optimum effectiveness in primary care.

## Introduction

 Physical activity (PA) counseling is an effective approach to increase PA levels among patients in primary care.^1-3^ The current World Health Organization (WHO) guidelines on PA recommend that people with chronic medical conditions should participate in at least 150–300 min/wk of moderate-intensity aerobic PA or at least 75–150 min/wk of vigorous-intensity aerobic PA or an equivalent combination, as well as at least 2 times/week muscle-strengthening and at least 3 times/week of multicomponent activities.^4^ Meeting the recommended levels of PA decreases the risk of major non-communicable diseases and premature death.^5-8^

 Practices and characteristics of PA counseling vary among different settings.^9-11^ A variety of models (e.g., transtheoretical model) and theories (e.g., social cognitive theory) have been applied to understand the mechanisms of PA behaviors.^12-14^ In addition, several programs for promoting PA have been implemented, such as Exercise is Medicine (in the USA, Canada, Australia, Poland, Singapore) and Green Prescription (in New Zealand).^10^These programs help primary care providers to provide PA counseling.

 A systematic review by Orrow et al revealed that promoting PA in 12 patients may prompt one patient to become more active.^15^ This highlights the importance of improving the prevalence of PA counseling in primary care. However, epidemiological studies report a wide range of prevalence of PA counseling in various settings.^16,17^ This limits the understanding of prevalence of PA counseling in primary care at the global level.

 This systematic review and meta-analysis aimed to summarize and evaluate the prevalence of PA counseling in primary care across various settings worldwide. In addition, the subgroup analyses aimed to calculate the pooled prevalence of PA counseling by sex and medical condition. Determining the current prevalence of PA counseling can help to understand current practices and set realistic goals to increase the prevalence.

## Materials and Methods

 The systematic review protocol was prospectively developed and registered with PROSPERO (registration number: CRD42021284570). This systematic review follows the reporting guidelines of the 2020 Preferred Reporting Items for Systematic Reviews and Meta-Analyses (PRISMA) statement.^18,19^

###  Information sources and search strategy

 Five databases, comprising CINAHL Complete, Embase, Medline (via the PubMed interface), PsycInfo, and Web of Science, were searched from database inception to 28^th^ November 2021. The updated search was performed at the time of manuscript revision (the updated search covered articles published between 28^th^ November 2021 and 28^th^ August 2023). The search strategy was based on the PICO mnemonic, which included population (any), intervention (physical activity counseling in primary care), comparison (none), and outcome (prevalence), as applicable. All the authors reviewed and finalized the search terms, which were adapted for each database (Supplementary file 1, Table S1). A filter was applied to obtain only English articles. The search results from each database were imported into an Endnote X9 reference manager (Thomson Reuters, Toronto, ON, Canada).

###  Eligibility criteria

 The inclusion criteria were publication in English andepidemiological studies that reported the prevalence of PA counseling in a primary care setting. The exclusion criteria were duplicates, unpublished studies, conference abstracts, expert opinion excerpts, review articles (i.e., systematic, scoping, and narrative reviews), protocols, and trial registrations (Supplementary file 1, Table S2).

###  Study selection 

 The lead author (AW) screened the titles and abstracts. Uncertainties were resolved by discussion with another author (SC). Two authors (AW and SL) then read the full-text articles and identified each as “Yes”, “No”, or “Maybe”. The level of agreement was determined based on the percent agreement and Cohen’s kappa (ĸ) using an online statistic calculator (GraphPad by Dotamics: https://www.graphpad.com/quickcalcs/kappa1/), with ĸ values of 0–0.20, 0.21–0.39, 0.40–0.59, 0.60–0.79, 0.80–0.90, and > 0.90 indicating no agreement, minimal agreement, weak agreement, moderate agreement, strong agreement, and almost perfect agreement, respectively.^20^ Any discrepancies between the two authors (“Yes”/“Maybe”, “No”/“Maybe”, “Maybe”/“Maybe”, or “Yes”/“No”) were resolved by discussion with another author (CL).

###  Data extraction

 The lead author (AW) extracted data using a data extraction form developed by the review team in Microsoft Word (Microsoft Inc., Redmond, WA, USA). The following information was extracted from each article: article title, name of first author, year of publication, country of study, study design, study participants and setting, characteristics and contents of PA counseling, and prevalence of PA counseling with 95% confidence interval (CI), if available. Another author (SL) cross-checked the data extraction forms, and any disagreements were resolved by consensus.

###  Quality assessment of individual studies

 The quality of each study was assessed by two authors (AW and SL) using the Joanna Briggs Institute (JBI) critical appraisal checklist for studies reporting prevalence data. The appraisal tool consists of nine questions with four standard answers (“Yes”, “No”, “Unclear”, or “Not applicable”). Based on the judgment of reviewers, the result for each study is “Include”, “Exclude”, or “Seek further information”.^21,22^

###  Data synthesis

 The data from the data extraction forms, encompassing both qualitative data (e.g., characteristics and content of PA counseling) and quantitative data (e.g., prevalence of PA counseling), were synthesized and presented in a narrative summary. For studies reporting multiple values of prevalence, the most recent prevalence was used in the narrative summary.

 Meta-analysis was conducted in R version 4.2.3 (R Foundation for Statistical Computing, Vienna, Austria) using the meta package.^23^ The pooled prevalence was calculated based on the number of participants who received PA counseling divided by the entire number of eligible participants. The reviewers contacted the corresponding author of each study via email to clarify unclear or missing numbers. If the corresponding authors did not respond within 2 weeks, the reviewers calculated the missing numbers based on the available data or, if this was not possible, excluded the study from the meta-analysis. Statistical heterogeneity was explored using the Cochran’s Q test (chi-square statistics) and quantified by I^2^. Owing the heterogeneity among the studies, a random-effects model was used to calculate the pooled prevalence, the prevalence of each medical condition and its 95% CIs. For studies reporting multiple values of prevalence, the most recent prevalence was included in the meta-analysis. A funnel plot was created to evaluate potential publication bias.

## Results

###  Study selection

**Figure 1:**
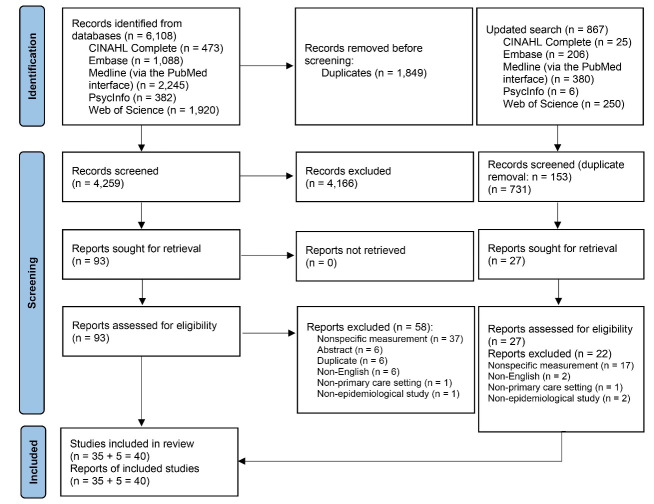
PRISMA flow diagram

 A total of 6975 articles were retrieved from the five databases, and 2002 duplicates were excluded. After screening the titles and abstracts, a further 4897 articles were excluded. The full-texts of the remaining 120 articles were then independently reviewed by two reviewers; their percentage agreement was 96.67% (116 out of 120 articles) and ĸ was 0.927 (standard error: 0.035, 95% CI: 0.859 to 0.995). Finally, 40 articles (representing 40 studies) were included in the analysis (Figure 1).

###  Study characteristics and quality assessment of studies

 The 40 included studies were published between 1992 and 2022. They were conducted in 17 countries: USA (n = 11), Australia (n = 5), Brazil (n = 3), Germany (n = 3), Barbados (n = 2), Lithuania (n = 2), New Zealand (n = 2), Poland (n = 2), UK (n = 2), Belgium (n = 1), Canada (n = 1), China (n = 1), Jamaica (n = 1), Spain (n = 1), Sweden (n = 1), Switzerland (n = 1), and Tanzania (n = 1). Four studies addressed PA counseling by direct observation or reviewing videotapes of doctor–patient visits.^24-27^ The number of eligible participants ranged from 14 to 90 240 (Table 1). The quality assessment scores (JBI scores) of the included studies ranged from 5 to 9 out of 9: 5/9 (n = 6), 6/9 (n = 12), 7/9 (n = 10), 8/9 (n = 7), and 9/9 (n = 5) (Table 1 and Supplementary file 1, Table S3).

**Table 1:** Study characteristics

**First author and year of publication (country)**	**JBI checklist**	**Data collection**	**Participants **	**Characteristics of PA counseling **	**Prevalence**
Adams et al 2010^28^ (Barbados)	6/9	Chart audit	343 charts of patients aged ≥ 40 with HT from public and private clinics	Recording exercise advice in patient’s chart	153/343 = 44.6%
Adams et al 2011^29^ (Barbados)	6/9	Chart audit	253 charts of patients aged ≥ 40 with DM from public and private clinics	Recording exercise advice in patient’s chart	124/253 = 49.0%
Ahmed et al 2017^30^ (USA)	6/9	Household interview surveys in 2000, 2005, and 2010	Adults aged ≥ 18 who were able to perform PA (without physical disability) and visited a doctor or other health care provider in the past 12 months (n = 23,656 in 2000; n = 26 152 in 2005; n = 21 905 in 2010)	“During the past 12 months, did a doctor or other health professional recommend that you begin or continue to do any type of exercise or physical activity?”	2000 (n = 23 656): 22.9% (95% CI 22.0 to 23.8) 2005 (n = 26 152): 30.4% (95% CI 29.7 to 31.0) 2010 (n = 21 905): 33.6% (95% CI: 32.8 to 34.4)
Barbosa et al 2017^31^ (Brazil)	8/9	Face-to-face interview	785 patients aged ≥ 20 with HT 823 patients aged ≥ 20 with DM with or without HT	“Has any health provider of the Family Health Strategy ever counseled you to modify (improve) your physical activity habits? (Yes/No)”	HT: 406/785 = 51.7% DM: 475/823 = 57.7%
Bovier et al 2007^32^ (Switzerland)	7/9	Face-to-face assessment and interview of the participating physician to analyze the last 20 medical records of patients who visited the physician’s office	186 community-based primary care physicians with 3684 patient records Records of patients with DM (n = 350, however 345 were eligible for PA counseling) and pre-DM (n = 181) who had a follow-up in the last 12 months were analyzed	Assessing the adherence to recommended standards of diabetes care, including promotion of daily PA	DM: 273/345 = 79.1% Pre-DM: 108/181 = 59.7%
Croteau et al 2006^33^ (New Zealand)	8/9	National postal survey	8291 adults (61% response rate)	Asking whether a doctor or practice nurse advised PA or gave a Green Prescription (written and verbal PA prescription scheme) in the past 12 months	PA advice: 1046/8291 = 12.6% Green Prescription: 235/8,291 = 2.8%
Daly et al 2015^34^ (New Zealand)	9/9	Telephone interview	265 patients with DM (out of 308 sampled patients = 96% of the sampled patients) seen by participating primary health care nurses	“Did you give advice about diet or physical activity?” Details of PA counseling were asked about, such as advice on increasing PA level, walking, mobility-limited activities, swimming, joining a gym or exercise class	PA advice: 175/265 = 66.0% Green Prescription: 11/265 = 4.2%
Davis-Ajami et al 2021^35^ (USA)	7/9	Questionnaire	1039 pre-DM adults aged ≥ 20 with overweight/obesity (BMI ≥ 25 kg/m^2^) Among 1039 participants, 798 participants received lifestyle advice (241 participants did not receive lifestyle advice)	Being advised to increase PA or exercise	Exercise advice (total participants: n = 1,039): 67.6% Exercise advice (only participants receiving lifestyle advice: n = 798): 87.9%
Desai et al 2002^36^ (USA)	8/9	Chart review	90 240 people with obesity (BMI ≥ 27 kg/m^2^) and/or HT and with/without mental conditions	Evidence of receipt of exercise counseling in the past 2 years Exercise counseling included needs for regular PA, benefits of PA, and methods for increasing PA	Overall: 88.5% No mental disorder: 88.7% Psychiatric disorder: 88.5% Substance use: 86.3% Psychiatric disorder and substance use: 85.7%
Eakin et al 2007^37^ (Australia)	6/9	Self-reported survey	Of 2,478 participants, 1999 participants (80.67%) visited a GP at least once in the last 12 months	“Did you receive any advice from your doctor about exercise or physical activity?”	483/1999 = 24.2%
Edward et al 2020^24^ (Tanzania)	7/9	Direct observation of patient consultation	Of 69 new patients aged ≥ 30, 14 were diagnosed with HT	Advising patients with HT to increase PA	3/14 = 21.43%
Egede et al 2002^38^ (USA)	7/9	Self-reported survey	Adults aged ≥ 18: 9,496 adults with DM and 150 493 adults without DM PA counseling was assessed among adults with a BMI ≥ 25 kg/m^2^ who had a checkup in the past 12 months	Receiving counseling about regular PA during at least one checkup in the past 12 months	DM (n = 875): 67.4% (95% CI 63.2 to 71.7) No DM (n = 10154): 36.0% (95% CI: 34.6 to 37.4)
Eldemire-Shearer et al 2009^39^ (Jamaica)	6/9	Face-to-face interview	738 adults aged ≥ 50 who visited health centers/hospitals/primary care services	Being advised about PA	24.5%
Flocke et al 2004^25^ (USA)	7/9	Direct observation of patient visits by research nurses Recall of health behavior advice was assessed by using an exit questionnaire	2,670 adults aged ≥ 18 who visited their family physicians and completed a patient exit survey	Exercise advice by family physicians	Direct observation: 603/2670 = 22.6% Patient recall 260/603 = 43.1%
Foss et al 1996^40^ (UK)	5/9	Self-administered questionnaire	2,676 adults aged ≥ 18 with mild to moderate HT (71% of participants recalled receiving lifestyle advice, so the number of participants who received lifestyle advice was 1900)	Advice on exercise	722/1900 = 38.0%
Gabrys et al 2015^17^ (Germany)	7/9	Retrieved data from two cross-sectional health interview and examination surveys (1997–1999 and 2008–2011)	Adults aged 18–64: M = 2891 and F = 3078 in the 1997–1999 survey; M = 2,789 and F = 3149 in the 2008–2011 survey	Self-reported PA counseling provided by a physician in the last 12 months	1997–1999: F = 9.3%; M = 11.1%; with DM = 10.8%; without DM = 11.1%; with HT = 14.1%; without HT = 10.6%; with CHD = 17.7%; without CHD = 10.8%; with cancer = 9.0%; without cancer = 11.1% 2008–2011: F = 7.7%; M = 9.4%; with DM = 29.8%; without DM = 8.6%; with HT = 16.8%; without HT = 8.1%; with CHD = 24.0%; without CHD = 10.9%; with cancer = 14.0%; without cancer = 9.3%
Geerling et al 2019^41^ (Australia)	5/9	Online questionnaire and hard-copy questionnaire completed by participants	381 adults aged ≥ 18 with type 2 DM	Using counseling techniques (each one of the 14 behavior change techniques) for PA	279/381 = 73.2% (receiving general advice – PA is important)
Gowin et al 2009^26^ (Poland)	6/9	Direct observation of consultation and medical record review for preventive procedures in the past year	450 adults aged ≥ 40 (F = 267 and M = 183) who visited 113 GPs (four consecutive patients were observed per GP)	11 preventive procedures, including PA counseling	37/450 = 8.2% F: 23/267 = 8.6% M: 14/183 = 7.6%
Hinrichs et al 2011^42^ (Germany)	6/9	Telephone interview	1937 older adults aged ≥ 65 who used to participate in a cohort study (7 years before this study) (310 participants were excluded due to incomplete data or advice to rest (did not recommend exercise)); 1,627 participants (F = 854 and M = 773) were included in the analysis	Advice to get regular exercise in the past 12 months	534/1627 = 32.8% F = 30.0% M = 36.0%
Hu et al 2021^43^ (China)	8/9	In-person questionnaire	454 patients with chronic conditions	“Did the physician provide PA advice to you just now?” If the participants were advised about PA, they were asked about whether they received advice on frequency, intensity, duration, and type.	87/454 = 19.2% (8/87 = 9.2% received all four components)
Johansson et al 2005^44^ (Sweden)	6/9	Postal questionnaire	6734 patients aged 18–79 who visited GPs at primary care centers (4163 participants responded to the question about exercise advice)	Advice on lifestyle habits, including physical exercise	677/4163 = 16.3%
Juré et al 2022^45^ (Belgium)	8/9	Chart review	3055 patients (F = 2310 and M = 745) aged ≥ 18 with chronic venous disease from 253 GPs (out of 3,103 sampled patients = 98.4% of the sampled patients)	Advice on PA	1,328/3055 = 43.5% F: 994/2310 = 43.0% M: 334/745 = 44.8%
Klumbiene et al 2006^46^ (Lithuania)	6/9	Mailed questionnaire from three surveys in 2000, 2002, and 2004	2049 adults (F = 1156 and M = 893) aged 20–64 with overweight and obesity who visited GPs in the past 12 months based on three surveys	“During the last year (12 months) have you been advised to increase your physical activity?”	F = 19.2% M = 15.9%
Kriaucioniene et al 2019^47^ (Lithuania)	6/9	Mailed questionnaire from eight surveys in 2000, 2002, 2004, 2006, 2008, 2010, 2012, and 2014	5867 adults aged 20–64 with overweight and obesity who visited GPs in the past 12 months based on eight surveys (2000: n = 699; 2002: n = 680; 2004: n = 670; 2006: n = 705; 2008: n = 738; 2010: n = 883; 2012: n = 779; and 2014: n = 713)	“During the last year (12 months) have you been advised by a GP to increase your physical activity?”	2000: 11.9% (95% CI: 9.6 to 14.1) 2002: 13.8% (95% CI: 11.3 to 16.3) 2004: 11.7% (95% CI: 9.4 to 14.1) 2006: 12.7% (95% CI: 10.3 to 15.1) 2008: 18.0% (95% CI: 15.3 to 20.8) 2010: 17.2% (95% CI: 14.9 to 19.6) 2012: 15.3% (95% CI: 12.8 to 17.8) 2014: 11.6% (95% CI: 9.2 to 13.9) Overall (2000–2014): 14.2% (95% CI: 13.3 to 15.1)
Lau et al 2013^48^ (USA)	5/9	Self-reported surveys in 2005 and 2007	Young adults aged 18–26 who responded to California Health Interview Survey in 2005 (n = 3670) and 2007 (n = 3,621); 2955 participants in 2005 responded to a question regarding exercise counseling (there was no question regarding exercise counseling in the 2007 survey)	“Did a health provider give you information about how much or what kind of exercise you get?”	Overall = 22.0% F = 24.5% M = 19.1%
Martínez-Gómez et al 2013^49^ (Spain)	8/9	Computer-assisted telephone interview with a structured questionnaire and two home visits for physical examination, biological sample (blood and urine) collection, and dietary history	12 985 adults aged ≥ 18 (1034 were excluded or missing); 11 951 participants were included	“Have you ever been counseled by your physician or nurse to do PA, in particular, walking for at least 30 min several days a week?”	5,591/11,951 = 46.2% (95% CI: 45.0 to 47.4%) F (n = 6,851): 50.2% (95% CI: 48.6 to 51.8) M (n = 6,191): 42.1% (40.4 to 43.8)
Nguyen et al 2011^50^ (USA)	6/9	Face-to-face interview from 2002–2006 (5 surveys)	1787 Mexican-American adults (F = 1,126 and M = 661) aged ≥ 18 with obesity (BMI ≥ 30 kg/m^2^) and a usual health care provider	“Has a doctor or other health care professional ever advised you to exercise more?”	Overall: 988/1,787 = 55.9% F: 655/1126 = 58.2% M: 333/661 = 50.4%
Ory et al 2006^27^ (USA)	5/9	Videotape (doctor–patient encounter) review	423 older adults aged ≥ 65 who were seen by 36 doctors at three primary care sites	Doctor–patient discussion on PA or nutrition or both PA and nutrition	PA only: 39.2% Both PA and nutrition: 22.0% Total PA discussion: 61.2%
Robertson et al 2011^51^ (Australia)	5/9	Computer-assisted telephone interview	1261 adults aged ≥ 18 (one eligible person per household)	Recommendations on doing some exercise/PA or increasing exercise/PA by a health professional in the past 12 months; the follow-up questions included questions on type of health professional and type of PA	PA recommendations by any health professional: 311/1,261 = 24.7% PA recommendations by GP: 225/1,261 = 17.8% Walking was the most common type of PA recommended by GPs: 175/225 = 77.8%
Dos Santos et al 2021^52^ (Brazil)	9/9	Face-to-face interview	779 adults (F = 544 and M = 235) aged ≥ 18 living in urban areas who visited basic health units (excluded a person who used the basic health unit for the first time)	“During the last year (12 months), any time you were at the basic health unit, did you receive PA counseling during the consultation by a health professional (advice, tips, or guidance on PA to change/improve your health)?”	Overall: 335/779 = 43.0% (95% CI: 39.5 to 46.4%) F: 43.6% M: 41.7%
Short et al 2016^53^ (Australia)	7/9	Online questionnaire	1799 adults aged ≥ 18	PA recommendations by a GP in the past 12 months, including type (i.e., aerobic PA, resistance-based PA, flexibility, balance, non-specific PA, cannot remember) and amount (duration and frequency)	328/1799 = 18.2% Received specific advice: 253/328 = 77.1% Most common types: aerobic PA: 150/253 = 59.3%; resistant PA: 34/253 = 13.4%; flexibility: 29/253 = 11.5%; balance: 11/253 = 4.4% Received specific amount of PA: 136/253 = 53.8%
Shuval et al 2014^54^ (USA)	5/9	Questionnaire	157 adults aged 40–79	General PA counseling in the past 12 months; components (5As: ask, advise, agree, assist, and arrange)	General PA counseling: 84/157 = 53.5% Ask: 5/157 = 3.2% Advise - verbal: 71/157 = 45.2%; written: 22/157 = 14.0% Agree: 54/157 = 34.4% Assist – overcoming barriers: 22/157 = 14.0%; identifying community resources and social support: 16/157 = 10.2% Arrange: 18/157 = 11.5%
Silagy et al 1992^55^ (UK)	8/9	Mailed questionnaire	4941 adults aged 35–64 who visited GPs in the past 12 months and subsequently attended for a health check	"In the last 12 months has a doctor or nurse advised you to take more exercise/stop smoking/drink less alcohol/change your diet or lose weight?"	PA advice: 222/4941 = 4.5%
Sinclair et al 2008^56^ (Canada)	7/9	Questionnaire	1562 adults aged ≥ 18 who reported having a regular family physician	“In visits to your usual family doctor [health care provider], how often were the following subjects discussed with you: advice on healthy eating and advice on appropriate exercise for you.” The analysis was performed according to two categories (often/always and never/rarely)	Overall prevalence of “often/always” discussed exercise = 42.0%
Smith et al 2019^57^ (Australia)	7/9	Case note review (10–20 consecutive patients visited 365 GPs)	6512 patients (F = 3,512 and M = 3,000) with HT who visited GPs	PA prescription	Overall: 2518/6512 = 38.7% F: 1325/3512 = 37.7% M: 1193/3000 = 39.8%
de Souza et al 2022^58^ (Brazil)	9/9	Face-to-face interview	779 adults (F = 544 and M = 235) aged ≥ 18 who visited primary care units	“During the past year (12 months), in a visit to the healthcare unit, did you receive physical activity counseling while in consultation with a healthcare professional (advice, tips or orientation on physical activity or exercise)?”	335/779 = 43.0%
Tiffe et al 2021^59^ (Germany)	6/9	Face-to-face interview	665 participants aged 30-79 without cardiovascular disease	Advice by a physician to increase PA	52.1%
Wee et al 1999^60^ (USA)	9/9	In-person survey	9711 adults aged ≥ 18 who had a medical check-up in the previous year; 9299 participants responded to a question regarding having exercise counseling provided by a physician	“During your last (medical) check-up, did the doctor recommend that you begin or continue to do any type of exercise or PA?"	Overall = 34.0% F = 33.0% M = 34.0%
Znyk et al 2022^61^ (Poland)	9/9	Face-to-face interview	896 adults aged ≥ 18 who visited primary care	“Has the family doctor ever talked to you about physical activity and exercise?”	Overall: 355/896 = 39.6% Overweight/obesity: 198/402 = 49.2%
Zwald et al 2019^62^ (USA)	7/9	Interview	11 062 adults aged ≥ 20; 8410 participants were eligible (utilizing health care services in the past year) for the analysis	“To lower your risk for certain diseases, during the last 12 months have you ever been told by a doctor or health professional to increase your PA or exercise?”	Overall (n = 8410): 42.9% (95% CI: 40.8 to 44.9) DM (n = 1570): 69.8% (95% CI: 66.5 to 72.8) HT (n = 3447): 56.5% (95% CI: 53.8 to 59.2) Obesity (n = 3390): 63.0% (95% CI: 60.3 to 65.7)

CI, confidence interval; BMI, body mass index; CHD, coronary heart disease; DM, diabetes mellitus; F, female; GP, general practitioner; HT, hypertension; JBI, Joanna Briggs Institute; M, male; PA, physical activity.

###  Prevalence of physical activity counseling 

**Figure 2:**
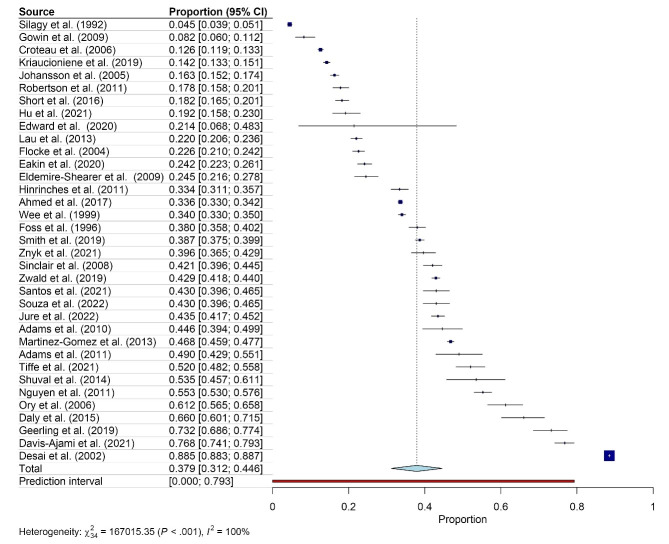
Pooled proportion of physical activity counseling

 The meta-analysis of 35 studies with a total of 199 830 participants showed that the pooled prevalence of PA counseling in primary care was 37.9% (95% CI: 31.2 to 44.6) (Figure 2 and Supplementary file 1, Table S4). Five studies were not included in the meta-analysis because it did not report the overall prevalence of PA counseling of the entire study participants. For example, they reported the prevalence in females and males separately.

**Figure 3:**
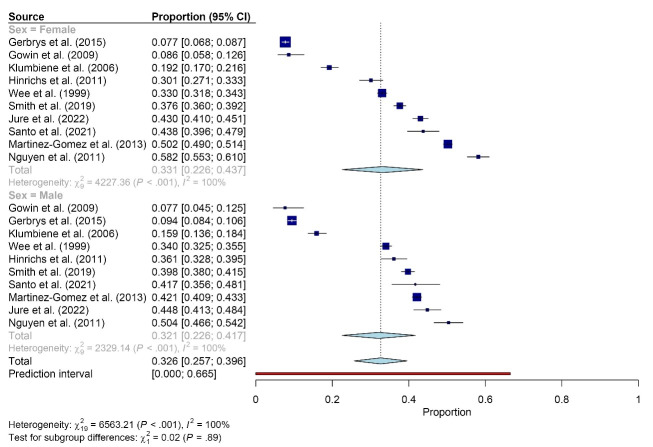
Pooled proportion of physical activity counseling by sex

 The pooled prevalence of PA counseling in primary care by sex was 33.1% (95% CI: 22.6 to 43.7) for females (n = 10; 25,255 participants) and 32.1% (95% CI: 22.6 to 41.7) for males (n = 10; 19,283 participants) (Figure 3 and Supplementary file 5, Table S5).

**Figure 4:**
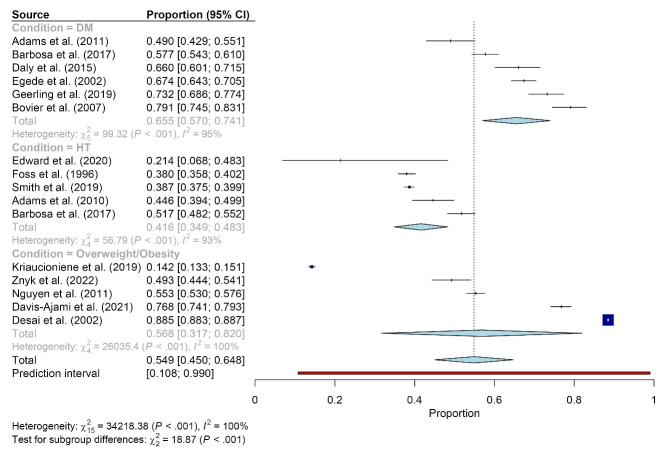
Pooled proportion of physical activity counseling by medical condition. Abbreviations: DM, diabetes mellitus; HT, hypertension

 The pooled prevalence of PA counseling in primary care was 65.5% (95% CI: 57.0 to 74.1; n = 6; 2942 participants) among people with diabetes mellitus (DM), 41.6% (95% CI: 34.9 to 48.3; n = 5; 9554 participants) among people with hypertension (HT), and 56.8% (95% CI: 31.7 to 82.0; n = 5; 99 335 participants) among people with overweight or obesity (Figure 4 and Supplementary file 6, Table S6).

###  Heterogeneity of studies and potential publication bias

**Figure 5:**
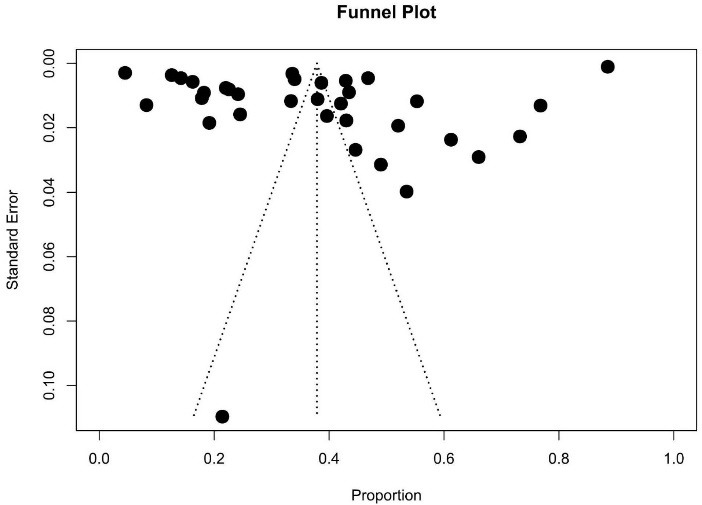
Funnel plot

 There were high levels of heterogeneity among studies. The I^2^ of the overall meta-analysis of 35 studies was 100% (P < 0.001) (Figure 2). The meta-analyses by sex and medical condition showed high levels of heterogeneity for both sexes (I^2^ = 100%, *P* < 0.001) (Figure 3) and medical conditions (DM: I^2^ = 95%, *P* < 0.001; HT: I^2^ = 93%, *P* < 0.05; and overweight or obesity: I^2^ = 100%, *P* < 0.001) (Figure 4). Figure 5 shows an asymmetrical funnel plot.

## Discussion

 This systematic review included 40 studies conducted in 17 countries from four out of six regions according to the WHO categories (Region of the Americas = 18 studies, European Region = 13 studies, Western Pacific Region = 8 studies, and African Region = 1 study).^63^ The overall prevalence of PA counseling calculated from 35 studies (199 830 participants) was 37.9%. By sex, the prevalence was 33.1% for females (10 studies; 25 255 participants) and 32.1% for males (10 studies; 19,283 participants). By medical condition, the prevalence among people with DM, HT, and overweight/obesity was 65.5% (6 studies; 2942 participants), 41.6% (5 studies; 9554 participants), and 56.8% (5 studies; 99 335 participants), respectively.

 Regarding the quality of individual studies based on the JBI critical appraisal checklist for studies reporting prevalence data, there were some issues. Most of the studies had unclear information about the appropriateness of sampling methods (20/40 studies, 50.0%), the adequacy of sample sizes (32/40 studies, 80.0%), and the adequacy of response rates (29/40 studies, 72.5%). This suggests the possibility of selection bias and non-response bias in many studies.^64^ Therefore, the results of this systematic review should be interpreted with caution based on these biases.

 The prevalence of PA counseling varied across various settings from low (4.5%) to high (88.5%). The previous systematic review by Hall et al reported the prevalence of PA screening in primary care ranging from 2.4% to 100% and the prevalence of PA advice in primary care ranging from 0.6% to 100%.^65^ However, the aforementioned systematic review included different types of studies (e.g., cross-sectional studies, qualitative studies) and sources of information on PA counseling (e.g., reported by health professionals and patients).^65^ In the present systematic review, primary studies that reported the prevalence of PA counseling based on estimates from health care providers were excluded from the pooled results in order to avoid overestimation. The previous systematic review did not perform meta-analysis.^65^ The present systematic review reported the pooled prevalence based on meta-analysis of primary studies.

 The meta-analyses revealed greater prevalence of PA counseling in females compared to males. This observation highlights the disparity in practices between these two population groups. One potential explanation is that primary care providers prioritize females as a higher-risk demographic for insufficient PA. The global data also supports that females were more likely to be physically inactive compared to males.^66-68^ Regarding medical conditions, the meta-analyses demonstrated higher prevalence of PA counseling among individuals with DM, HT, and overweight/obesity compared to the overall prevalence. PA is regarded as an essential treatment and preventive strategy for these medical conditions.^69-71^ Consequently, the emphasis on PA counseling in primary care was particularly pronounced within these populations.

 High levels of heterogeneity were identified in all meta-analyses. One potential reason for this is that there were different data collection and outcome measurement methods among the individual studies, which may impact the reported prevalence of PA counseling in different studies. Another potential reason was that there is a lack of standard guidelines for PA counseling in primary care. The 2020 WHO guidelines on PA state the recommendations for clinical populations; however, this report did not indicate specific guidelines for clinical practices.^4^ Although various approaches to PA counseling in primary care have been published,^11,72-76^ the development of standard guidelines for PA counseling in primary care is recommended.

 Most of the included studies in this review (39/40, 97.5%) were conducted in three regions (Region of the Americas, European Region, and Western Pacific Region).^63^ This trend was similar to the findings on research productivity in the field of PA research that showed a large proportion of publications have been produced in these three regions.^77,78^ Our finding may reflect the influence of research productivity and the contribution of studies on PA counseling in primary care in the various regions. In the other regions of the world, there is a need to highlight PA research, which may improve insights into PA counseling in primary care.

 This systematic review has several strengths. First, it focused on a specific setting, primary care. Second, it involved meta-analyses of the primary care population and subgroup populations (by sex and medical condition). Third, studies that reported the prevalence of PA counseling estimated by health care providers were excluded from the pooled results to avoid overestimation. A major limitation of the review is that the differences in PA counseling and outcome measurement methods among primary studies may affect the pooled prevalence and cause high levels of heterogeneity in the meta-analyses.

## Conclusion

 The prevalence of PA counseling in primary care is low (37.9%) and varies between studies (from 4.5% to 88.5%). PA counseling is more common among people with DM, overweight/obesity, and HT. Due to the high levels of study heterogeneity and different PA counseling approaches, there is a need to develop practical guidelines for PA counseling in primary care.

## Competing Interests

 The authors declared no competing interest.

## Ethical Approval

 Not applicable.

## Supplementary Files


Supplementary file 1 contains Table S1-S6.
