# Supplementary material for: Prevalence of physical activity counseling in primary care: A systematic review and meta-analysis
Source: Health Promot Perspect. 2023 Dec 16;13(4):254–66. doi: 10.34172/hpp.2023.31 (PMC10790122; doi:10.34172/hpp.2023.31)
Supplement: Supplementary file 1 — contains Table S1-S6. [file hpp-13-254-s001.pdf]

Wattanapisit et al, **Health Promotion Perspectives**, 2023, 13(4), 254-S1.
doi: 10.34172/hpp.2023.31
https://hpp.tbzmed.ac.ir

Table S1. Search terms

PICO	Description	Search Terms
**Population**	Any	-
**Intervention**	Physical activity counseling in primary care	"physical activity" OR "physical activit*" OR exercise* OR "physical exercise*" OR fitness OR "physical fitness"<br>AND<br>counselling OR counseling OR promotion OR promot* OR advice OR advise OR prescription OR prescribe<br>AND<br>"primary care" OR "primary health care" OR "family medicine" OR "family practice" OR "general practice"
**Comparison**	None	-
		AND
**Outcome**	Prevalence	prevalence OR rate OR proportion

Table S2. Inclusion and exclusion criteria

Component	Inclusion criteria	Exclusion criteria
**Type of study**	• Epidemiological studies	• Experimental studies<br>• Clinical trials<br>• Qualitative studies
**Type of article**	• Research or original articles	• Review articles (systematic, scoping, narrative reviews)<br>• Protocols<br>• Unpublished articles<br>• Conference abstracts<br>• Expert opinion excerpts<br>• Trial registrations
**Population**	• All age groups	• None
**Intervention**	• PA/exercise counseling provided by primary care providers<br>• PA/exercise prescription provided by primary care providers	• Combined lifestyle or obesity counseling without separated information on PA counseling<br>• PA/exercise referrals
**Setting**	• Primary care settings<br>• Primary health care settings	• Specialized clinics/settings<br>• Exercise facilities/centers
**Outcome**	• Prevalence of PA counseling reported on providers' practices or chart reviews (the number of PA counseling sessions divided by the number of primary care users)<br>• Prevalence of PA counseling reported by primary care users (the number of PA counseling sessions divided by the number of primary care users)	• Prevalence based on self-estimation (e.g., always providing PA counseling, counsel more half of patients)<br>• Prevalence of PA/exercise referrals

PA = physical activity.

Table S3. Quality assessment of studies

**First author and year of publication**	1.Was the sample frame appropriate to address the target population?	2. Were study participants sampled in an appropriate way?	3. Was the sample size adequate?	4. Were the study subjects and the setting described in detail?	5. Was the data analysis conducted with sufficient coverage of the identified sample?	6. Were valid methods used for the identification of the condition?	7. Was the condition measured in a standard, reliable way for all participants?	8. Was there appropriate statistical analysis?	9. Was the response rate adequate, and if not, was the low response rate managed appropriately?
Adams et al. 2010[28]	Y	U	U	Y	Y	Y	Y	Y	U
Adams et al. 2011[29]	Y	U	U	Y	Y	Y	Y	Y	U
Ahmed et al. 2017[30]	Y	U	U	Y	Y	Y	Y	Y	U
Barbosa et al. 2017[31]	Y	Y	U	Y	Y	Y	Y	Y	Y
Bovier et al. 2007[32]	Y	U	U	Y	Y	Y	Y	Y	Y
Croteau et al 2006[33]	Y	Y	U	Y	Y	Y	Y	Y	Y
Daly et al. 2015[34]	Y	Y	Y	Y	Y	Y	Y	Y	Y
Davis-Ajami et al. 2021[35]	Y	Y	U	Y	Y	Y	Y	Y	U
Desai et al. 2002[36]	Y	Y	Y	Y	Y	Y	Y	Y	U
Eakin et al. 2007[37]	Y	U	U	Y	Y	Y	Y	Y	U
Edward et al. 2020[24]	Y	Y	U	Y	Y	Y	Y	Y	U
Egede et al. 2002[38]	Y	Y	U	Y	Y	Y	Y	Y	U
Eldemire-Shearer et al. 2009[39]	Y	U	U	Y	Y	Y	Y	Y	U
Flocke et al. 2004[25]	Y	Y	U	Y	Y	Y	Y	Y	U
Foss et al. 1996[40]	Y	U	U	Y	Y	Y	U	Y	U
Gabrys et al. 2015[17]	Y	Y	U	Y	Y	Y	Y	Y	U
Geerling et al. 2019[41]	Y	U	U	U	Y	Y	Y	Y	U
Gowin et al. 2009[26]	Y	U	U	Y	Y	Y	Y	Y	U
Hinrichs et al. 2011[42]	Y	U	U	Y	Y	Y	Y	Y	U
Hu et al. 2021[43]	Y	U	Y	Y	Y	Y	Y	Y	Y
Johansson et al. 2005[44]	Y	U	U	Y	Y	Y	Y	Y	U
Jure et al. 2022[45]	Y	Y	U	Y	Y	Y	Y	Y	Y
Klumbiene et al. 2006[46]	Y	U	U	Y	Y	Y	Y	Y	U
Kriaucioniene et al. 2019[47]	Y	U	U	Y	Y	Y	Y	Y	U
Lau et al. 2013[48]	Y	U	U	Y	U	Y	Y	Y	U
Martínez-Gómez et al. 2013[49]	Y	Y	U	Y	Y	Y	Y	Y	Y
Nguyen et al. 2011[50]	Y	U	U	Y	Y	Y	Y	Y	U
Ory et al. 2006[27]	U	U	U	Y	Y	Y	Y	Y	U
Robertson et al. 2011[51]	U	U	U	Y	Y	Y	Y	Y	U
Santos et al. 2021[52]	Y	Y	Y	Y	Y	Y	Y	Y	Y
Short et al. 2016[53]	Y	Y	U	Y	Y	Y	Y	Y	U
Shuval et al. 2014[54]	U	U	U	Y	Y	Y	Y	Y	U
Silagy et al. 1992[55]	Y	Y	Y	Y	Y	Y	Y	Y	U
Sinclair et al. 2008[56]	Y	Y	U	Y	Y	Y	Y	Y	U
Smith et al. 2019[57]	Y	Y	U	Y	Y	Y	Y	Y	U
Souza et al. 2022[58]	Y	Y	Y	Y	Y	Y	Y	Y	Y
Tiffe et al. 2021[59]	Y	U	U	Y	Y	Y	Y	Y	U
Wee et al. 1999[60]	Y	Y	Y	Y	Y	Y	Y	Y	Y
Znyk et al. 2022[61]	Y	Y	Y	Y	Y	Y	Y	Y	Y

First author and year of publication	1.Was the sample frame appropriate to address the target population?	2. Were study participants sampled in an appropriate way?	3. Was the sample size adequate?	4. Were the study subjects and the setting described in detail?	5. Was the data analysis conducted with sufficient coverage of the identified sample?	6. Were valid methods used for the identification of the condition?	7. Was the condition measured in a standard, reliable way for all participants?	8. Was there appropriate statistical analysis?	9. Was the response rate adequate, and if not, was the low response rate managed appropriately?
Zwald et al. 2019[62]	Y	Y	U	Y	Y	Y	Y	Y	U

N = no; N/A = not applicable; U = unclear; Y = yes.

Table S4. Overall pooled prevalence of physical activity counseling (n = 35)

1	Adams et al. 2010[28] (Barbados)	HT only	153/343 = 44.6%
2	Adams et al. 2011[29] (Barbados)	DM only	124/253 = 49.0%
3	Ahmed et al. 2017[30] (USA)		2010 (n = 21,905): 33.6% (95%CI 32.8 to 34.4): 7,361/21,905 = 33.6%
4	Croteau et al 2006[33] (New Zealand)		PA advice: 1,046/8,291 = 12.6%
5	Daly et al. 2015[34] (New Zealand)	DM only	PA advice: 175/265 = 66.0%
6	Davis-Ajami et al. 2021[35] (USA)	Overweight and obesity	Exercise advice: 702/1,039 = 67.6%
7	Desai et al. 2002[36] (USA)	Obesity and/or HT	Overall: 88.5%: 79,863/90,240 = 88.5%
8	Eakin et al. 2007[37] (Australia)		483/1,999 = 24.2%
9	Edward et al. 2020[24] (Tanzania)	HT only	3/14 = 21.43%
10	Eldemire-Shearer et al. 2009[39] (Jamaica)		24.5%: 181/738 = 24.5%
11	Flocke et al. 2004[25] (USA)		Direct observation: 603/2,670 = 22.6%
12	Foss et al. 1996[40] (UK)	HT only	722/1,900 = 38.0%
13	Geerling et al. 2019[41] (Australia)	DM only	279/381 = 73.2% (receiving general advice – PA is important)
14	Gowin et al. 2009[26] (Poland)		37/450 = 8.2%
15	Hinrichs et al. 2011[42] (Germany)		534/1,627 = 32.8%
16	Hu et al. 2021[43] (China)	Chronic conditions	87/454 = 19.2%
17	Johansson et al. 2005[44] (Sweden)		677/4,163 = 16.3%
18	Jure et al. 2022[45] (Belgium)	Chronic venous disease	1,328/3,055 = 43.5%
19	Kriaucioniene et al. 2019[47] (Lithuania)	Overweight and obesity	Overall (2000-2014): 14.2% (95%CI 13.3 to 15.1): 834/5,867 = 14.2%
20	Lau et al. 2013[48] (USA)		Overall = 22.0%: 651/2,955 = 22.0%
21	Martínez-Gómez et al. 2013[49] (Spain)		5,591/11,951 = 46.2% (95% CI 45.0 to 47.4%)
22	Nguyen et al. 2011[50] (USA)	Obesity	Overall: 988/1,787 = 55.9%
23	Ory et al. 2006[27] (USA)		Total PA discussion: 61.2%: 259/423 = 61.2%
24	Robertson et al. 2011[51] (Australia)		PA recommendations by GP: 225/1,261 = 17.8%
25	Santos et al. 2021[52] (Brazil)		Overall: 335/779 = 43.0% (95%CI 39.5 to 46.4%)
26	Short et al. 2016[53] (Australia)		328/1,799 = 18.2%
27	Shuval et al. 2014[54] (USA)		General PA counseling: 84/157 = 53.5%
28	Silagy et al. 1992[55] (UK)		PA advice: 222/4,941 = 4.5%
29	Sinclair et al. 2008[56] (Canada)		Overall = 42.0%: 657/1,562 = 42.0%
30	Smith et al. 2019[57] (Australia)		Overall: 2,518/6,512 = 38.7%
31	Souza et al. 2022[58] (Brazil)		335/779 = 43.0%
32	Tiffe et al. 2021[59] (Germany)		347/665 = 52.1%
33	Wee et al. 1999[60] (USA)		Overall = 34.0%: 3,162/9,299 = 34.0%
34	Znyk et al. 2022[61] (Poland)		Overall: 355/896 = 39.6%
35	Zwald et al. 2019[62] (USA)		Overall (n = 8,410): 42.9% (95%CI 40.8 to 44.9): 3,608/8,410 = 42.9%

DM = diabetes mellitus; HT = hypertension; PA = physical activity.

Table S5. Pooled prevalence of physical activity counseling by sex: female (n = 10) and male (n = 10)

**Female**		
1	Gabrys et al. 2015[17] (Germany)	F = 7.7%: 243/3,149 = 7.7%
2	Gowin et al. 2009[26] (Poland)	F: 23/267 = 8.6%
3	Hinrichs et al. 2011[42] (Germany)	F = 30.0%: 257/854 = 30.0%
4	Jure et al. 2022[45] (Belgium)	F: 994/2,310 = 43.0%
5	Klumbiene et al. 2006[46] (Lithuania)	F = 19.2%: 222/1,156 = 19.2%
6	Martínez-Gómez et al. 2013[49] (Spain)	F (n = 6,851): 50.2% (95%CI 48.6 to 51.8): 3,440/6,851 = 50.2%
7	Nguyen et al. 2011[50] (USA)	F: 655/1,126 = 58.2%
8	Santos et al. 2021[52] (Brazil)	F: 43.6%: 238/544 = 43.6%
9	Smith et al. 2019[57] (Australia)	F: 1,325/3,512 = 37.7%
10	Wee et al. 1999[60] (USA)	F = 33.0%: 1,811/5,486 = 33.0%
**Male**		
1	Gabrys et al. 2015[17] (Germany)	M = 9.4%: 263/2,789 = 9.4%
2	Gowin et al. 2009[26] (Poland)	M: 14/183 = 7.6%
3	Hinrichs et al. 2011[42] (Germany)	M = 36.0%: 279/773 = 36.0%
4	Jure et al. 2022[45] (Belgium)	M: 334/745 =44.8%
5	Klumbiene et al. 2006[46] (Lithuania)	M = 15.9%: 142/893 = 15.9%
6	Martínez-Gómez et al. 2013[49] (Spain)	M (n = 6,191): 42.1% (40.4 to 43.8): 2,607/6,191 = 42.1%
7	Nguyen et al. 2011[50] (USA)	M: 333/661 = 50.4%
8	Santos et al. 2021[52] (Brazil)	M: 41.7%: 98/235 = 41.7%
9	Smith et al. 2019[57] (Australia)	M: 1,193/3,000 = 39.8%
10	Wee et al. 1999[60] (USA)	M = 34.0%: 1,297/3,813 = 34.0%

F = female; M = male.

Table S6. Pooled prevalence of physical activity counseling by medical conditions: diabetes mellitus (n = 6), hypertension (n = 5), and overweight/obesity (n = 5)

**DM**		
1	Adams et al. 2011[29] (Barbados)	124/253 = 49.0%
2	Barbosa et al. 2017[31] (Brazil)	DM: 475/823 = 57.7%
3	Bovier et al. 2007[32] (Switzerland)	DM: 273/345 = 79.1%
4	Daly et al. 2015[34] (New Zealand)	PA advice: 175/265 = 66.0%
5	Egede et al. 2002[38] (USA)	DM (n = 875): 67.4% (95CI% 63.2 to 71.7): 590/875 = 67.4%
6	Geerling et al. 2019[41] (Australia)	279/381 = 73.2% (receiving general advice – PA is important)
**HT**		
1	Adams et al. 2010[28] (Barbados)	153/343 = 44.6%
2	Barbosa et al. 2017[31] (Brazil)	HT: 406/785 = 51.7%
3	Edward et al. 2020[24] (Tanzania)	3/14 = 21.43%
4	Foss et al. 1996[40] (UK)	722/1,900 = 38.0%
5	Smith et al. 2019[57] (Australia)	Overall: 2,518/6,512 = 38.7%
**Overweight/obesity**		
1	Davis-Ajami et al. 2021[35] (USA)	Exercise advice: 702/1,039 = 67.6%
2	Desai et al. 2002[36] (USA)	Overall: 88.5%: 79,863/90,240 = 88.5%
3	Kriaucioniene et al. 2019[47] (Lithuania)	Overall (2000-2014): 14.2% (95%CI 13.3 to 15.1): 834/5,867 = 14.2%
4	Nguyen et al. 2011[50] (USA)	Overall: 988/1,787 = 55.9%
5	Znyk et al. 2022[61] (Poland)	Overweight/obesity: 198/402 = 49.2%

DM = diabetes mellitus; HT = hypertension; PA = physical activity.
